# Examining the association between early life social adversity and BMI changes in childhood: a life course trajectory analysis

**DOI:** 10.1111/ijpo.12063

**Published:** 2015-08-25

**Authors:** T. T. Morris, K. Northstone, L. D. Howe

**Affiliations:** ^1^ School of Geographical SciencesUniversity of BristolBristolUK; ^2^ School of Social and Community MedicineUniversity of BristolBristolUK; ^3^ MRC Integrative Epidemiology UnitUniversity of BristolBristolUK

**Keywords:** ALSPAC, Obesity, residential mobility, social adversity

## Abstract

**Background:**

A number of studies have found associations between multiple aspects of social adversity and obesity in childhood, yet this research has largely been limited to cross‐sectional data.

**Objectives:**

This study aimed to address this limitation by using life course trajectory methods to determine whether multiple aspects of social adversity in early childhood are associated with changes in body mass index (BMI) throughout childhood.

**Methods:**

Associations between multiple measures of social adversity from birth to 4 years and subsequent BMI trajectories to age 17 were examined in 7021 children in the Avon Longitudinal Study of Parents and Children.

**Results:**

Higher BMI throughout ages 12–17 were observed for children whose parents had separated, were exposed to frequent residential mobility or who experienced moderate or great household financial difficulty in early childhood. After adjustment for confounding variables, associations were attenuated but remained for both moderate (two moves) and high (≥3 moves) residential mobility (mean % difference in BMI at age 17 for children experiencing moderate and high residential mobility before age 4 compared with those experiencing no moves: 2.3; 95% CI: 0.5–4.2; *P* = 0.015 and 4.2; 95% CI: 1.4–7.0; *P* = 0.004, respectively).

**Conclusions:**

Associations between BMI and social adversity in childhood are present but largely explained by background socioeconomic position. However, there remain small but important differences between the BMI of children who are exposed to frequent residential mobility in early childhood after adjustment for socioeconomic and other confounders.

## Introduction

The prevalence of childhood obesity has continued to rise at an alarming rate throughout much of the world 1, 2 and many studies have identified social factors that may lead to obesogenic environments 3. Given the links between obesity and a broad range of short‐ and long‐term negative health outcomes 4, it is important that potential social contributors to obesity risk in childhood are identified and understood. There is now considerable evidence that low socioeconomic position (SEP) is associated with an increased risk of obesity in children 5, 6 and that this inequality begins at an early age 7.

Other family social factors may also lead to a greater risk of childhood obesity, but these have been less well studied. There is evidence that obesity is more prevalent among children who have experienced adverse life events which disrupt the living environment such as residential moves, financial hardship, parental divorce, parental separation and family death 8, 9, 10, 11, 12, 13, 14. It is hypothesized that adverse events can lead to stress 15 and reduced parental availability, resources and monitoring 16, which in turn may increase obesity risk through negative changes in parenting behaviour and attitudes 17, 18. However, many existing studies have utilized cross‐sectional approaches and therefore cannot examine how early any associations emerge or whether they change throughout childhood.

A better understanding of when in the life course these associations emerge and how they vary with age may inform stronger design of preventative interventions for tackling childhood obesity. In order to increase this understanding, we conduct a longitudinal study using appropriate life course trajectory methods to determine whether a range of adverse events in early childhood are associated with changes in body mass index (BMI) throughout the first 17 years of the life course. Life course trajectory models allow a more detailed interpretation of associations than cross‐sectional models widely used as they can elucidate longitudinal development and permit full use of available data. The data we use come from a large prospective birth cohort study from the UK.

## Methods

### Study population

Participants were children from the Avon Longitudinal Study of Parents and Children (ALSPAC). Pregnant women were eligible to enrol if they had an expected date of delivery between April 1991 and December 1992 and were resident in the (former) Avon Health Authority area in South West England (for full details of the cohort profile and study design, see 19 and 20). Supporting Information Fig. S1 shows the available sample for our study. From the core sample of 14 676 children, 7021 have full data on BMI, social adversity measures and all covariates. The ALSPAC cohort is largely representative of the UK when compared with 1991 Census data; with slight under‐representation in ethnic minorities, single parent families and those living in rented accommodation. Ethical approval for the study was obtained from the ALSPAC Ethics and Law Committee and the Local Research Ethics Committees. Please note that the study website contains details of all the data that are available through a fully searchable data dictionary 21.

### Exposures

Residential mobility was classified as the number of household moves occurring in the first 4 years of the study child's life. Household moves were self‐reported by the mother in questionnaires, clinic visits, or by using address change forms supplied by ALSPAC with each questionnaire that could be returned via post. The detailed recording of address changes provides an extremely detailed and temporally accurate source of residential moves that had occurred in the child's life. Because of low numbers of participants moving more than three times, the total number of residential moves was recoded to a categorical variable for analysis with four values: ‘no moves’, ‘1 move’, ‘2 moves’ and ‘3 + moves’. This cut‐off of three or more moves is consistent with that used widely in previous residential mobility research 8.

At four time points (32 weeks gestation; 8, 21 and 33 months), mothers were asked to report how difficult it was for them to afford each of food, clothing, heating, rent/mortgage and other essentials for their child. Individual item responses were recoded from a 4‐point Likert scale and summed to provide an overall financial difficulty score across all items for the four time points where a value of 0 represented no financial difficulty and a value of 15 represented maximum financial difficulty. These scores were then categorized as ‘no financial difficulty’ (0), ‘some financial difficulty’ 1, 2, 3, 4, ‘moderate financial difficulty’ 5, 6, 7, 8, 9 and ‘great financial difficulty’ 10, 11, 12, 13, 14, 15.

At 8, 21, 33 and 47 months, mothers and their partners were asked to report whether their respective partners had died or separated from them. Responses to these two questions were recoded to create binary variables indicating the occurrence of parental death or separation by the child's fourth birthday.

Mothers and their partners were asked at 33 and 47 months to report whether either of the parents had lost their job. Responses from both parents were recoded to create a binary variable indicating whether a parent had experienced a job loss by the time the child was 4 years old.

### Outcome

Weight and height data were used from a variety of sources including health visitor records, parental reports and measurements taken at clinic visits. Four measurements were taken from routine health visitor records (as part of standard UK childcare) at around 2, 10, 21 and 48 months; mothers were asked to report their child's height and weight regularly from 3 years; clinics were carried out at 4, 8, 12 and 18 months for a subset of 10% of participants and at 7, 8, 9, 10, 11, 12, 13, 15 and 17 years for all participants. BMI was calculated as weight (kg) divided by height (m) squared. All measurements prior to age 4 were excluded from the analysis to permit modelling from the measurement of exposure time point.

### Confounders

A range of confounding variables were obtained from self‐reported questionnaires prior to age 4 including highest maternal education at pregnancy (categorized as below O level [exams taken at completion of compulsory school attendance at age 16], O level, A level [exams taken in post‐compulsory schooling at age 18] and university degree or above); family disposable income; mother's smoking habits during the first trimester (categorized as yes/no); parity as reported at 18 weeks gestation; home ownership status (categorized as owned/mortgaged, rented from council or housing association, and rented privately/other); mother's cohabitation with partner; mother's pre‐pregnancy BMI; and ethnicity.

### Statistical analyses

Implausible measurements of BMI (>4 SD [standard deviations] from the mean for each age and gender) were examined and removed if inconsistent with the child's other growth measures (this accounted for <0.1% of all measurements). Previous research has demonstrated that the accuracy of measurements varies between routine and parent‐reported measurements but that measurements from research clinics and routine child healthcare records have similar accuracy 22, 23, and therefore a binary indicator of measurement source (health care/research measurement vs. maternal reported) was included in all models.

Individual BMI trajectories were estimated using mixed‐effects multilevel models with measurement occasions at level 1 nested within individuals at level 2. Mixed‐effects models permit individual variation in growth trajectories as random effects allow each individual participant to have unique intercepts and slopes. They utilize all available data from eligible participants under the assumption of data missing at random, therefore minimizing the impact of attrition by including all participants with one or more BMI measurements regardless of missing data at other time points. BMI was modelled on the natural log scale because it is positively skewed; the coefficients presented in all results are back‐transformed from the log scale and can be interpreted as the percentage difference in BMI compared with the baseline category of each exposure. Fractional polynomials were used to allow for non‐linearity of the BMI trajectory 24. Fractional polynomials involve considering a wide range of polynomials, and can therefore be used to model a broader range of curve shapes than is possible with standard polynomial models. We considered 16 functions of age and tested all sets of two or three functions; the best‐fitting model, selected based on the likelihood value and differences between observed and predicted measurements, had the form: BMI_ij_ = (β_0_ + u_0j_ + e_0ij_) + (β_1_ + u_1j_)(ln(age)_ij_) + (β_2_ + u_2j_)(age*ln(age)_ij_) + (β_3_ + u_3j_)(age^2^*ln(age)_ij_) + β_4_(male_j_) + β_5_(male_j_*ln(age)_ij_) + β_6_(male_j_*age*ln(age)_ij_) + β_7_(male_j_*age^2^*ln(age)_ij_) + (β_8_ + e_1ij_)(measurement_source_ij_) + e_1ij_(age_months_ij_), where for person j at measurement occasion i; βs represents fixed effect coefficients, u_0_–u_3_ indicate person‐specific random effects for the intercept and linear, quadratic and cubic age terms, respectively, and e_1_ represents the occasion‐specific residuals.

All analyses were conducted using STATA v 13 (StataCorp, College Station, TX, USA) using the runmlwin command 25, which calls MLwiN version 2.30 [www.cmm.bristol.ac.uk/MLwiN/index.shtml]. Measurements predicted by the multilevel models were compared with actual measurements to assess model fit.

## Results

### Participant characteristics

BMI measures were available for 3592 boys and 3429 girls who also had full data on all covariates. Table 1 displays descriptive characteristics of the sample. A very small number of children experienced parental death (n = 30; 0.43%) and a minority experienced either a parent losing a job (n = 1060; 15.10%) or parents separating (n = 878; 12.51%). The majority of children experienced no residential moves between birth and 4 years (n = 4134; 58.88%), with decreasing proportions of children experiencing an increased number of moves during this time period (1 move n = 2033; 28.96%; 2 moves n = 564; 8.03%; 3+ moves n = 290; 4.13%). Financial difficulties were distributed more evenly with large proportions of children experiencing no (n = 2687; 38.27%) and some (n = 2874; 40.93%) financial difficulties, and decreasing proportions experiencing moderate (n = 1247; 17.76%) and great (n = 213; 3.03%) financial difficulties.

**Table 1:** Characteristics of participants

Characteristic		n (%)
Exposure		
Parent lost job	No	5961 (84.90)
	Yes	1060 (15.10)
Parent died	No	6991 (99.57)
	Yes	30 (0.43)
Parents separated	No	6143 (87.49)
	Yes	878 (12.51)
Number of household moves	0	4134 (58.88)
	1	2033 (28.96)
	2	564 (8.03)
	3 +	290 (4.13)
Financial difficulties	None	2687 (38.27)
	Some	2874 (40.93)
	Moderate	1247 (17.76)
	Great	213 (3.03)
Confounders		Mean (SD)
Family income		233.19 (105.49)
Maternal education	Lower than O level	1569 (22.35)
	O level	2528 (36.01)
	A level	1802 (25.67)
	Degree or higher	1122 (15.98)
Home ownership	Owned/mortgaged	5738 (81.73)
	Council/HA rented	708 (10.08)
	Private rented/other	575 (8.19)
Maternal smoking during pregnancy	No	5628 (80.16)
	Yes	1393 (19.84)
Parental cohabitation	No	345 (4.91)
	Yes	6676 (95.09)
Ethnicity	White	6786 (96.65)
	Non‐white	235 (3.35)
Sex	Girls in BMI model	3429 (48.84)
	Boys in BMI model	3592 (51.16)
Parity	0	3207 (45.68)
	1	2560 (36.46)
	2 +	1254 (17.86)
Pre‐pregnancy BMI		22.97 (3.78)
Number of BMI measurements	Median	9
	IQR	5–12

BMI, body mass index; HA, housing association; IQR, interquartile range.

### Model fit

Differences between observed BMI and BMI predicted by the mixed‐effects model remained reasonably small throughout all years indicating good model fit (Supporting Information Table S1).

### Social influences on BMI trajectories

Assessing exposures with BMI trajectories independently, there was evidence of an association between parental job loss in early childhood and BMI at ages 15 and 16: children whose parents had experienced a job loss had a mean BMI of 1.1% (95% CI: 0.0–2.2) and 1.2% (95% CI: 0.1–2.3), respectively, higher than those whose parents' employment remained stable (Supporting Information Table S2). Parental separation was associated with a higher BMI from ages 7 to 17 with associations monotonically growing in strength with increasing age culminating in a BMI difference of 1.7% (95% CI: 0.2–3.2) at age 17.

There was evidence of an association between multiple house moves in early childhood and increased BMI from age 12. Associations were strongest among children who had moved three or more times, culminating in a 4.3% (95% CI: 1.8–6.7) difference in BMI at age 17 compared to those who had not moved, while the difference at 17 between those moving twice and those not moving was 2.7% (95% CI: 1.0–4.4). There was also weak evidence for increased BMI between ages 4 and 8 among those who had moved three or more times. Children from households experiencing financial difficulties had a higher BMI than children from financially secure households. This association increased throughout late childhood and showed a dose–response effect of 5.6% (95% CI: 2.8–8.4) higher in the great group, 2.7% (95% CI: 1.4–4.0) higher in the moderate group and 1.0% (95% CI: 0.0–1.9) higher in some group at age 17.

**Figure 1:**
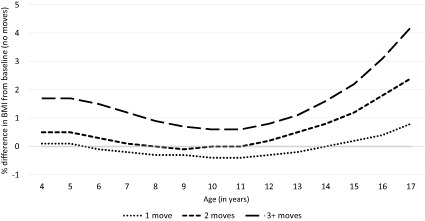
BMI trajectories of residential mobility groups.

Adjustment for covariates (Table 2) attenuated associations. After adjustment, children whose parents had separated had a BMI of 1.1% (95% CI: 0.2–2.0) higher at age 4 than those whose parents remained together, but this diminished by age 9. Children who moved twice or three or more times still had a higher BMI from ages 16 and 15, respectively, than children who did not move. At age 17, these associations represented 2.3% (95% CI: 0.5–4.2) and 4.2% (95% CI: 1.4–7.0) higher BMIs compared to children who did not move. Figure 1 displays the differences in mean BMI trajectories throughout the study period across mobility categories in graphical format. The attenuation of these associations was driven largely by the influence of housing tenure in our models; other covariates accounted for only modest reduction of results. Given the potential distinction between parental death and other exposures from the child's perspective, we reran the results excluding parental death and this did not change our results. To test if the observed mobility associations were driven by moves to better or worse environments, we additionally ran a model accounting for changes between neighbourhood deprivation quintiles; however, this made negligible difference to results and therefore is not presented.

**Table 2:** Adjusted predicted % difference in BMI from baseline group for each family disruption measure

Age	Lost job	Parent died	Parents separated	No. of residential moves			Financial difficulties		
				1 move	2 moves	3+ moves	Some difficulty	Moderate difficulty	Great difficulty
4	−0.1 (−0.8 to 0.6)	0.6 (−3.3 to 4.6)	1.1 (0.2–2)*	0.1 (−0.5 to 0.7)	0.5 (−0.5 to 1.5)	1.6 (0.2–3.1)*	−0.2 (−0.8 to 0.3)	−0.7 (−1.5 to 0.1)	−0.4 (−2.1 to 1.2)
5	−0.1 (−0.8 to 0.6)	0.6 (−3.3 to 4.6)	1.1 (0.2–2)*	0.1 (−0.5 to 0.7)	0.5 (−0.5 to 1.5)	1.6 (0.2–3.1)*	−0.2 (−0.8 to 0.3)	−0.7 (−1.5 to 0.1)	−0.4 (−2.1 to 1.2)
6	0.1 (−0.6 to 0.8)	0 (−4.0 to 4.1)	1.0 (0.2–1.9)*	−0.1 (−0.7 to 0.5)	0.2 (−0.7 to 1.2)	1.5 (0–2.9)*	−0.2 (−0.7 to 0.4)	−0.6 (−1.4 to 0.2)	−0.4 (−2.0 to 1.3)
7	0.2 (−0.5 to 0.9)	−0.4 (−4.7 to 3.9)	1.0 (0.1–1.9)*	−0.2 (−0.8 to 0.4)	0.1 (−1.0 to 1.1)	1.2 (−0.3 to 2.6)	0 (−0.6 to 0.6)	−0.3 (−1.1 to 0.5)	−0.4 (−2.0 to 1.3)
8	0.3 (−0.5 to 1.1)	−0.7 (−5.5 to 4.0)	1.1 (0.1–2)*	−0.3 (−1.0 to 0.4)	0 (−1.2 to 1.1)	0.9 (−0.7 to 2.5)	0.1 (−0.6 to 0.8)	0 (−0.9 to 0.9)	−0.3 (−2.2 to 1.5)
9	0.4 (−0.5 to 1.3)	−1.1 (−6.4 to 4.3)	1.1 (0–2.2)	−0.4 (−1.1 to 0.4)	−0.1 (−1.4 to 1.2)	0.7 (−1.1 to 2.5)	0.2 (−0.5 to 1.0)	0.3 (−0.7 to 1.3)	−0.2 (−2.3 to 1.8)
10	0.5 (−0.5 to 1.5)	−1.4 (−7.3 to 4.5)	1.1 (−0.1 to 2.3)	−0.4 (−1.2 to 0.4)	−0.1 (−1.5 to 1.3)	0.6 (−1.4 to 2.5)	0.4 (−0.4 to 1.2)	0.5 (−0.6 to 1.6)	−0.1 (−2.4 to 2.1)
11	0.5 (−0.5 to 1.6)	−1.7 (−8.0 to 4.6)	1.1 (−0.2 to 2.3)	−0.4 (−1.2 to 0.5)	0 (−1.5 to 1.5)	0.6 (−1.5 to 2.7)	0.5 (−0.4 to 1.3)	0.7 (−0.4 to 1.9)	0.1 (−2.3 to 2.5)
12	0.6 (−0.5 to 1.7)	−2.0 (−8.5 to 4.5)	1.0 (−0.3 to 2.3)	−0.3 (−1.2 to 0.6)	0.2 (−1.3 to 1.7)	0.8 (−1.4 to 2.9)	0.6 (−0.3 to 1.5)	0.9 (−0.3 to 2.1)	0.3 (−2.2 to 2.8)
13	0.6 (−0.4 to 1.7)	−2.3 (−8.8 to 4.2)	1.0 (−0.4 to 2.3)	−0.2 (−1.1 to 0.7)	0.4 (−1.1 to 1.9)	1.1 (−1.1 to 3.2)	0.6 (−0.3 to 1.5)	1.0 (−0.2 to 2.2)	0.6 (−1.8 to 3.1)
14	0.7 (−0.4 to 1.7)	−2.6 (−9.1 to 3.8)	0.9 (−0.4 to 2.2)	−0.1 (−0.9 to 0.8)	0.8 (−0.7 to 2.3)	1.6 (−0.6 to 3.7)	0.6 (−0.2 to 1.5)	1.0 (−0.2 to 2.2)	1.0 (−1.4 to 3.5)
15	0.7 (−0.4 to 1.8)	−3.0 (−9.4 to 3.5)	0.8 (−0.6 to 2.1)	0.2 (−0.7 to 1.0)	1.2 (−0.3 to 2.7)	2.2 (0.1–4.4)*	0.6 (−0.2 to 1.5)	1.0 (−0.2 to 2.2)	1.5 (−1.0 to 4.0)
16	0.7 (−0.5 to 1.8)	−3.3 (−10.3 to 3.6)	0.6 (−0.8 to 2.1)	0.4 (−0.5 to 1.4)	1.7 (0.1–3.3)*	3.1 (0.7–5.5)**	0.6 (−0.3 to 1.5)	0.9 (−0.4 to 2.2)	2.1 (−0.6 to 4.8)
17	0.7 (−0.7 to 2.0)	−3.7 (−11.9 to 4.5)	0.5 (−1.3 to 2.2)	0.7 (−0.4 to 1.8)	2.3 (0.5–4.2)*	4.2 (1.4–7.0)**	0.5 (−0.6 to 1.6)	0.6 (−0.9 to 2.2)	2.7 (−0.5 to 5.9)

Results represent % change in BMI from the baseline group of each exposure at each age. Baseline groups are no event for job loss, parental death, parental separation and household moves, and no difficulty for financial difficulty. Results are adjusted for family income, maternal education, home ownership, maternal smoking during pregnancy, parental cohabitation, ethnicity, sex, parity, maternal pre‐pregnancy BMI and family disruption events. *, ** indicate significant differences compared to baseline values (*P ≤ 0.05; **P ≤ 0.01).

## Discussion

### Main findings and interpretations

Our longitudinal random effects approach considering a range of indicators of social adversity provides limited evidence that disruptions to the social environment in the first 4 years are associated with changes in BMI throughout childhood. BMI is higher among children whose families experience parental job loss, parental separation, two or three residential moves and financial difficulties. However, many of these associations are accounted for by family SEP. There does remain a positive association between residential mobility and BMI, observed at both the beginning and end of our study period, suggesting that regularly moving house may have immediate and long‐term negative effects on child's health. Children who moved three or more times in the first 4 years of life already had a BMI of 1.7% higher at age 4 than those who had not moved, indicating the presence of underlying BMI differences between high and low mobility groups. Additionally, despite a slight weakening in differences between ages 6 and 10, this increased from age 11 onwards creating a fanning out of mobility BMI trajectories with age. There was evidence for a dose–response relationship as the increase in BMI at age 17 for children who moved twice compared to those who did not move was just over two‐thirds (2.7%) that of the increase for those who moved three or more times (4.2%). There was also evidence for a positive association between parental separation and BMI as has been previously identified by other studies 11, 13, although this association was small and diminished with time indicating only short‐term effects of separation on BMI. Parental death was associated with lower BMI throughout the whole of childhood and adolescence but there was insufficient power to reliably determine associations (n = 30). This finding requires replication in larger studies.

Our results support arguments that increased residential mobility has negative effects on health outcomes consistent with other studies 8, 26. Moving just once may provide only a brief disturbance to the living environment whereas moving regularly may mean sustained disruption to networks and environment that could lead to negative health implications. Our results back this suggestion as the effects of moving once are marginal in comparison to not moving. While it was beyond the scope of our study, future research may investigate if particular groups of children exposed to multiple residential moves are resistant to excess increase in BMI.

However, our findings conflict with the only comparable UK study that we are aware of: Brown *et al*. 27 found no association between childhood mobility and BMI in a Scottish cohort. The difference in findings may be in part due to differences in modelling strategy or the classification of exposure (moves through all of childhood vs. only early childhood). It is plausible that the first 4 years of life are a critical period for exposure to family disruption in the development of increased adiposity and that this may have led to the difference in findings. Household disruption is socially patterned 28 and harmful to the health and development of children 29, meaning that there is potential for high residential mobility to exacerbate and reinforce socioeconomic inequalities in obesity. Given the recent rapid increase in the proportion of families with young children renting privately from 9% in 2003/2004 to over 21% in 2012/2013 and so subject to increased residential mobility 30, these findings are of importance as they may help identify children at greater risk of obesity. The inclusion of home ownership in our models suggests that it is disruption of the home environment rather than underlying factors associated with tenure that is driving this change in BMI.

The main strengths of this study are its longitudinal approach for examining the dynamic associations of disruption to early life home environments with adiposity over time and the use of a large representative sample with many repeat measurements. The use of longitudinal multilevel modelling permitted us to examine early life course trajectories of BMI regardless of missing data, varying ages at measurement and numbers of measurements in children. Our results are robust to consideration of a wide range of socioeconomic covariates known to influence BMI in early life and concurrent adverse life events, and are also not compromised by issues of selective migration in the same way as studies on adult residential mobility may be as children have little input on moving decisions. Nevertheless, limitations exist in our study which warrant discussion. Firstly, we do not account for disruptions occurring after 4 years of age and while this is difficult to avoid as it is necessary for modelling purposes, it is possible that the observed effects in adolescence could be compounded by further changes to the living environment that are not captured by our study methodology. Secondly, it is also possible that changes in BMI during adolescence may be influenced by unobserved effects of puberty, but given our modelling strategy of utilizing exposures prior to 4 years to model trajectories we were not able to include such effects.

In conclusion, we have shown that for a number of disturbances to the early childhood home environment, associations with BMI in childhood are present but largely explained by background socioeconomic factors. However, there are small and important differences between the BMI of children who experience frequent residential moves in early childhood that remain after adjustment. While some residential moves are unavoidable, these results suggest that policies to reduce frequent residential mobility and increase housing security may have beneficial effects on child health in the UK.

## Conflict of Interest Statement

The authors declare no conflict of interest.

## Funding

At the time of writing, TTM and KN were funded by the UK Medical Research Council and the Wellcome Trust (Grant Ref. 102215/2/13/2) with core support from the University of Bristol. LDH is funded by a UK Medical Research Council Fellowship (G1002375) and the UK Economic and Social Research Council (ES/M010317/1) and works in a unit that receives funding from the University of Bristol and the UK Medical Research Council (MC_UU_12013/5 and MC_UU_12013/9). Research reported in this publication was supported by the National Institute on Aging of the National Institutes of Health under Award No. R01AG048835. The content is solely the responsibility of the authors and does not necessarily represent the official views of the National Institutes of Health.

## Supporting information


**Figure S1.** Flow chart showing ALSPAC data available for this study.
**Table S1.** Model fit of observed and predicted BMI values.
**Table S2.** Unadjusted predicted % difference in BMI from baseline group for each family disruption measure.
