# Supplementary material for: Examining the association between early life social adversity and BMI changes in childhood: a life course trajectory analysis
Source: Pediatr Obes. 2015 Aug 25;11(4):306–12. doi: 10.1111/ijpo.12063 (PMC4767691; doi:10.1111/ijpo.12063)
Supplement: Supplementary file 1 — Figure S1. Flow chart showing ALSPAC data available for this study. Table S1. Model fit of observed and predicted BMI values. Table S2. Unadjusted predicted % difference in BMI from baseline group for each family disruption measure. [file IJPO-11-306-s001.docx]

Examining the association between early life social adversity and BMI changes: supplementary information.

Figure S1: Flowchart showing ALSPAC data available for this study.

Missing data on key variables

(n = 2568)

Missing social adversity data

(n = 1039)

Missing BMI data

(n = 644)

Not alive at 1 year; triplets & quadruplets (n = 692)

Data on social adversity measures

(n = 12 301)

BMI data available

(n = 13 340)

Singletons and twins alive at 1 year

(n = 13 984)

The core ALSPAC sample

(n = 14 676)

Data on covariates

(n = 9 733)

Missing data on covariates

(n = 2712)

Complete cases: data on BMI, adversity, and covariates available

(n = 7 021)

Table S1: Model fit of observed and predicted BMI values

| Age range | Observations | Mean (SD) observed data | Mean (SD) predicted data | Mean (95% reference range) for difference between observed and predicted values |
| --- | --- | --- | --- | --- |
| 4-7 | 8109 | 15.85 (1.74) | 15.76 (1.34) | 0.09 (-1.79 to 1.97) |
| 7-10 | 13910 | 16.87 (2.41) | 16.87 (2.28) | 0.00 (-1.08 to 1.08) |
| 10-13 | 13963 | 18.79 (3.31) | 18.75 (3.15) | 0.04 (-1.21 to 1.29) |
| 13-17 | 10651 | 20.45 (3.44) | 20.46 (3.31) | -0.02 (-1.62 to 1.59) |

SD, standard deviation.

Table S2: Unadjusted predicted % difference in BMI from baseline group for each family disruption measure

| Age | Lost job | Parent died | Parents separated | Number of residential moves | | | Financial difficulties | | |
| --- | --- | --- | --- | --- | --- | --- | --- | --- | --- |
|  |  |  |  | 1 move | 2 moves | 3+ moves | Some difficulty | Moderate difficulty | Great difficulty |
| 4 | -0.2 (-0.9 to 0.4) | -1.0 (-4.7 to 2.7) | 0.6 (-0.1 to 1.4) | 0.0 (-0.5 to 0.5) | 0.3 (-0.5 to 1.2) | 1.7 (0.5 to 2.9)** | -0.4 (-0.9 to 0.1) | -0.6 (-1.2 to 0.1) | 0.2 (-1.2 to 1.6) |
| 5 | -0.2 (-0.9 to 0.5) | -1.0 (-4.7 to 2.7) | 0.6 (-0.1 to 1.4) | 0.0 (-0.5 to 0.5) | 0.3 (-0.5 to 1.2) | 1.7 (0.5 to 2.9)** | -0.4 (-0.9 to 0.1) | -0.6 (-1.2 to 0.1) | 0.2 (-1.2 to 1.6) |
| 6 | -0.1 (-0.7 to 0.6) | -1.8 (-5.6 to 2.0) | 0.7 (0.0 to 1.4) | -0.1 (-0.7 to 0.4) | 0.2 (-0.7 to 1.1) | 1.6 (0.4 to 2.8)** | -0.2 (-0.7 to 0.3) | -0.3 (-1.0 to 0.3) | 0.5 (-0.9 to 1.9) |
| 7 | 0.1 (-0.6 to 0.8) | -2.3 (-6.4 to 1.8) | 0.8 (0.0 to 1.6)* | -0.2 (-0.8 to 0.3) | 0.1 (-0.8 to 1.0) | 1.5 (0.2 to 2.8)* | 0.0 (-0.6 to 0.5) | 0.1 (-0.6 to 0.7) | 0.9 (-0.6 to 2.4) |
| 8 | 0.2 (-0.6 to 1.0) | -2.7 (-7.2 to 1.9) | 0.9 (0.1 to 1.8)* | -0.3 (-0.9 to 0.4) | 0.1 (-1.0 to 1.1) | 1.5 (0.1 to 2.9)* | 0.2 (-0.4 to 0.8) | 0.5 (-0.3 to 1.3) | 1.3 (-0.3 to 2.9) |
| 9 | 0.4 (-0.5 to 1.2) | -3.0 (-8.1 to 2.2) | 1.1 (0.1 to 2.0)* | -0.3 (-0.9 to 0.4) | 0.1 (-1.1 to 1.2) | 1.5 (-0.1 to 3.0) | 0.4 (-0.2 to 1.1) | 0.9 (0.1 to 1.8)* | 1.8 (0.0 to 3.6)* |
| 10 | 0.5 (-0.5 to 1.5) | -3.3 (-8.9 to 2.4) | 1.2 (0.1 to 2.2)* | -0.3 (-1.0 to 0.5) | 0.1 (-1.1 to 1.4) | 1.5 (-0.2 to 3.3) | 0.6 (-0.1 to 1.3) | 1.4 (0.4 to 2.3)** | 2.3 (0.3 to 4.2)* |
| 11 | 0.6 (-0.4 to 1.6) | -3.5 (-9.6 to 2.6) | 1.3 (0.2 to 2.4)* | -0.2 (-1.0 to 0.6) | 0.3 (-1.1 to 1.6) | 1.7 (-0.1 to 3.5) | 0.8 (0.0 to 1.6) | 1.8 (0.8 to 2.8)** | 2.8 (0.7 to 4.9)* |
| 12 | 0.8 (-0.3 to 1.8) | -3.7 (-10.0 to 2.6) | 1.4 (0.3 to 2.6)* | -0.1 (-0.9 to 0.7) | 0.5 (-0.9 to 1.9) | 1.9 (0.0 to 3.8)* | 0.9 (0.1 to 1.7) | 2.1 (1.1 to 3.2)*** | 3.3 (1.1 to 5.4)** |
| 13 | 0.9 (-0.2 to 1.9) | -4.0 (-10.3 to 2.3) | 1.5 (0.4 to 2.7)* | 0.0 (-0.8 to 0.8) | 0.8 (-0.6 to 2.2) | 2.2 (0.3 to 4.1)* | 1.0 (0.2 to 1.8) | 2.4 (1.3 to 3.4)*** | 3.8 (1.6 to 5.9)*** |
| 14 | 1.0 (-0.1 to 2.0) | -4.2 (-10.4 to 2.0) | 1.6 (0.4 to 2.8)** | 0.1 (-0.7 to 0.9) | 1.1 (-0.3 to 2.5) | 2.6 (0.7 to 4.5)** | 1.1 (0.3 to 1.9)** | 2.6 (1.6 to 3.6)*** | 4.2 (2.1 to 6.4)*** |
| 15 | 1.1 (0.0 to 2.2)* | -4.4 (-10.6 to 1.8) | 1.7 (0.5 to 2.8)** | 0.3 (-0.5 to 1.1) | 1.6 (0.2 to 3.0)* | 3.0 (1.1 to 5.0)** | 1.1 (0.3 to 1.9)** | 2.7 (1.7 to 3.8)*** | 4.7 (2.5 to 6.9)*** |
| 16 | 1.2 (0.1 to 2.3)* | -4.7 (-11.2 to 1.9) | 1.7 (0.4 to 3.0)** | 0.5 (-0.3 to 1.4) | 2.1 (0.6 to 3.6)** | 3.6 (1.5 to 5.7)*** | 1.1 (0.2 to 1.9)* | 2.8 (1.6 to 3.9)*** | 5.2 (2.8 to 7.6)*** |
| 17 | 1.3 (0.0 to 2.6) | -4.9 (-12.4 to 2.6) | 1.7 (0.2 to 3.2)* | 0.8 (-0.2 to 1.8) | 2.7 (1.0 to 4.4)** | 4.3 (1.8 to 6.7)*** | 1.0 (0.0 to 1.9)* | 2.7 (1.4 to 4.0)*** | 5.6 (2.8 to 8.4)*** |

Results represent % change in BMI from the baseline group of each exposure at each age. Baseline groups are no event for job loss, parental death, parental separation, and household moves, and no difficulty for financial difficulty. Each exposure is modelled independently of other exposures. *,**,*** indicate significant differences compared to baseline values (*p≤0.05; **p≤0.01; ***p≤0.001).
